# Effect of Zishenpingchan Granule on Neurobehavioral Manifestations and the Activity and Gene Expression of Striatal Dopamine D1 and D2 Receptors of Rats with Levodopa-Induced Dyskinesias

**DOI:** 10.1155/2014/342506

**Published:** 2014-11-12

**Authors:** Qing Ye, Xiao-Lei Yuan, Jie Zhou, Can-xing Yuan, Xu-ming Yang

**Affiliations:** ^1^Department of Neurology, Longhua Hospital Affiliated to Shanghai University of Traditional Chinese Medicine, Shanghai 200032, China; ^2^Department of Acupuncture Institute, Shanghai University of Traditional Chinese Medicine, Shanghai 201203, China

## Abstract

This study was performed to observe the effects of Zishenpingchan granule on neurobehavioral manifestations and the activity and gene expression of striatal dopamine D1 and D2 receptors of rats with levodopa-induced dyskinesias (LID). We established normal control group, LID model group, and TCM intervention group. Each group received treatment for 4 weeks. Artificial neural network (ANN) was applied to excavate the main factor influencing variation in neurobehavioral manifestations of rats with LID. The results showed that overactivation in direct pathway mediated by dopamine D1 receptor and overinhibition in indirect pathway mediated by dopamine D2 receptor may be the main mechanism of LID. TCM increased the efficacy time of LD to ameliorate LID symptoms effectively mainly by upregulating dopamine D2 receptor gene expression.

## 1. Introduction

Parkinson's disease (PD) is a common chronic progressive neurodegenerative disorder in middle-aged and elderly people. The main pathological characteristic of PD is degeneration of dopaminergic neurons in the substantia nigra compact. At present, levodopa is still the gold standard for treating motor symptoms of PD, but more than 50% of PD patients treated with levodopa develop levodopa-induced dyskinesias in long-term therapy (generally over 5 years) [1, 2]. The major clinical manifestations are chorea or athetoid involuntary movements, myotonia, or myoclonus. Torso and head-face are also involved. It has a serious impact on the life quality of PD patients. The development of LID is related to loss of dopaminergic neurons control in the substantia nigra-striatum and the pulsatile treatment of LD, but the exact mechanism is not clear. Most consider that LID is due to the imbalance between the direct and indirect pathways in basal ganglia and also due to dopamine receptor hypersensitivity [3, 4]. Therefore, it is important to clarify the mechanism of LD inducing LID and find prevention and control measures, so as to improve the symptoms and the life quality of PD's advanced stage. This study mainly focused on the effect of Zishenpingchan granule on neurobehavioral manifestations and the activity and gene expression of striatal dopamine D1 and D2 receptors of rats with LID and provided theoretical and experimental basis for further study.

## 2. Materials and Methods

### 2.1. Drugs

Zishenpingchan granule is an effective Chinese herbal drug of the old Shanghai famous specialist of TCM Jianhua Hu. It is composed of Shudihuang 15 g, Gouqi 15 g, Sangjisheng 20 g, Tianma 15 g, Jiangcan 10 g, Ezhu 15 g, Baishaoyao 20 g, Tiannanxing 15 g, Quanxie 3 g, and Wugong 3 g. And it was made into granule by Jiangyin Tianjiang Pharmaceutical Co., Ltd. (Lot. 0504312). Each bag weighs 4.8 g equivalent to 32 g of crude drug. We mixed 4 bags of Zishenpingchan granule (equivalent to adult dosage of a day, containing 128 g of crude drug) and dissolved it in 100 mL normal saline. Each 1 mL contains 1.28 g crude drugs. LD powder (Lot. SLD6382) and benserazide powder (Lot. SL06492) were provided by Sigma-Aldrich (Shanghai) Co., Ltd., America.

### 2.2. Animals

Male SD rats (SCXK 2003-0002), weighing 180–220 g each, were purchased from Shanghai Sippr BK Laboratory Animals Ltd.

### 2.3. Instruments and Reagents

Apomorphine (APO), 6-hydroxydopamine (6-OHDA), SCH23390, and Spiperone were obtained from Sigma-Aldrich (Shanghai) Co., Ltd., USA. 3H-SCH23390 (69.8 Ci/mmol) and 3H-Spiperone (14.1 Ci/mmol) were from Amersham Co., Ltd., USA. TaKaRa reserve transcription reagent and PCR reactions kit were provided by TaKaRa Biotechnology (Dalian) Co., Ltd., while DEPC was from SBS Company. Rat brain stereotaxis instrument (T0w-3A) was from the Second Military Medical University. Ultracentrifuge (LE80K) was from Beckman Company. Liquid scintillation luminescence numeration (Wallacl450) was from PerkinElmer Inc., USA. IQS Multicolor Real-Time PCR Detection System was from BIO-RAD, USA, and nucleic acid protein detector was from Eppendorf, Germany.

### 2.4. Preparation of PD Rat Model [5]

Adult Sprague-Dawley (SD) rats were anesthetized by 1% pentobarbital before they were fixed on the rat stereotaxis instrument referring to the brain stereotaxic atlas made by Bao and Shu [6] and we determined the 3D coordinate of right SNC and VTA. (1) SNC: it was behind the anterior fontanelle 4.8 mm, on the right side of the sagittal suture 2.0 mm, and under the dura 8.0 mm. (2) VTA: it was behind the anterior fontanelle 4.8 mm, on the right side of the sagittal suture 1.2 mm, and under the dura 8.2 mm. We punched holes by using dental drill and injected 6-OHDA 6 *μ*g into the two coordinates (soluble in water mass fraction of saline 0.2% ascorbic acid, concentration of 2 *μ*g/*μ*) with injection speed 1 *μ*L/min and needle retaining time of 10 min. After operation, we packed skull hole with the gelatin sponge, sutured the skin incision, and injected gentamicin and then put the rats back to the rearing cage after they had woken up. Behavioral test began at 2 weeks after operation, using intraperitoneal injection of Apomorphine (0.5 mg/kg), when rats were constantly turned to the left and the rotational number > 210 r/30 min is regarded as a successful model of PD. Normal control group rat model preparation: and then we injected normal saline into right SNC and VTA, respectively, using the method described above (only containing 0.2% ascorbic acid). Processing method is in the same place after the operation. The experiment was approved by the Experimental Animal Ethics Committee of Shanghai University of Traditional Chinese Medicine in China (Approval number 09047).

### 2.5. Preparation of LID Rat Model

PD rats with successful modeling were produced by intraperitoneally injecting LD (10 mg/kg) and benserazide (2.5 mg/mL, dissolved in sterile normal saline with 0.05% ethanol and 0.05% ascorbic acid) twice a day (10 mg/kg per day) for 4 weeks. And we screened out the LID model based on presence of AIM including stereotyped act and contralateral rotation [7] (specific provisions of AIM score > 20 as a successful model of the LID).

### 2.6. Determination of Neurobehavioral Manifestations

#### 2.6.1. Determination of AIM Scores

AIM was measured in each group once a week as described by Cao et al. [8]. The assessment of rats was done every 30 min immediately after injection of LD and benserazide and continued for 120 min. There were five sets of data, and the sum of all the data was the final evaluation results. We divided AMI score into 4 parts (fore leg, oral-facial region, axial, and movement) for evaluation. Each part was divided into 5 levels on the scale of zero to 4: zero for none; 1 for occasionally; 2 for frequently occurring; 3 for persistency but stimulation can make it stop; 4 for persistency and stimulation cannot make it stop. Theoretically, the highest AMI score of a rat after drug taken once is 64.

#### 2.6.2. Peak Dose Rotation [9]

On the 7th, 14th, 21st, and 28th mornings during the treatment, neurobehavioral manifestations and AMI score of each group were observed. After injection of LD, we recorded rotation every 5 min. The largest number of rotation was the peak dose rotation.

#### 2.6.3. Efficacy Time of LD [10, 11]

The observation of neurobehavioral manifestations was performed once a week. We recorded the contralateral rotation laps every 5 min after injection of LD within 2 hours. Then we calculated the total laps of rotation and obtained the average number of rotation laps every 5 min within 2 hours. And we took the time between the first 5 min the number of rotation laps increased to the half of the average number and the first 5 min the number decreased to the half of average after injection of LD as the efficacy time of LD.

### 2.7. Grouping and Drug Administration of Experimental Animals

Totally 22 PD rats of 42 rats were successfully modeled according to the determination of neurobehavioral manifestations. After 4 weeks of LD treatment, 16 successful LID rats were screened out and divided into LID group and TCM group, 8 rats each. Another 8 rats served as control group. LID group continued to get injection of LD and benserazide (10 mg/kg, twice a day). TCM group rats were given TCM (9 mL/kg, once a day, i.g.) on the basis of LID group. And rats in the control group were injected and given intragastric administration of NS for 4 weeks.

### 2.8. Rat Brain Tissue RNA Isolation

Rats were anesthetized by 1% sodium pentobarbital at 2 hours after the last administration. After decapitation, brain tissues were stripped in an ice bath and the bilateral midbrain nigra and caudate putamen brain tissue were accurately cut and transferred to the homogenizer, respectively. They were kept with 1 mL Trizol at room temperature for 5–10 minutes. Then they were transferred to doff tubes, added to 0.2 mL chloroform, and vibrated for 15 min. After 3 minutes' standing at 4°C room temperature, 4°C 12000 g (15000 rpm) for 15 min, supernatant was transferred to another doff tube and added to 0.5 mL isopropyl alcohol, still standing at 4°C for 15 min, 4°C 12000 g (15000 rpm) for 10 min. After removal of supernatant, we added 1 mL 95% ethanol for washing the precipitation, 4°C 8000 g (10000 rpm) for 5 min. Then we dried the deposition and added DEPC to dissolve, kept at 20°C.

### 2.9. Reverse Transcription

FQ-PCR was used with the primers (10D each pair): D1 mRNA: F (GCTATGCTGACTGGGCTGAC); R (TTTCAGGGATGCTGCCTCT); D2 mRNA: F (CACTCAGATGCTTGCCATTGTTC); R (GTGGGATGTTGCAATCACAGTGTA). Reaction conditions were as follows: at 37°C for 15 min to the reverse transcription reaction and at 85°C for 5 sec to the inactivation of reverse transcriptase reaction.

### 2.10. FQ-PCR Reaction Conditions

We measured the relative content of each mRNA by using quantitative PCR: 95°C, 0.05 min→*T*_*m*_: 0.2 min→72°C, 0.1 min→72°C, and 5 min (*T*_*m*_: B-action-STR 61.6°C, B-action SN 61.4°C, D1 57.0°C, and D2 60.0°C). According to the FQ-PCR indirect quantitative formula, the average relative content % = 2 average −  ΔΔCt, ΔΔCt = ΔCt  sample −  ΔCt control, ΔCt  sample = ΔCt  sample −  ΔCt internal control.

### 2.11. Preparation of Membrane Proteins

We prepared the membrane protein as described by Zhang et al. [12]. Rats were anesthetized by 1% sodium pentobarbital at 2 hours after the last administration. After decapitation, brain tissues were stripped on ice and the bilateral midbrain nigra and caudate putamen brain tissue were accurately cut, respectively. And we added precooling centrifugal buffer of 4°C and made it into homogenate in an ice bath. The precipitate was diluted in 1-2 mL trishydrochloric acid buffer (pH 7.4) and fully suspended to membrane protein suspension. The membrane protein level was measured by Coomassie brilliant blue method and adjusted to 1 g/L.

### 2.12. Determination of Dopamine Receptor Activity

Using the double compound tubes method (each sample was divided into 9 different concentrations of reaction tube, repeat test twice), we added marked ligands whose concentration was increasing by multiple (specific binding, 10, 20, 40, 60, 80, and 100 *μ*L; nonspecific binding 20, 60, and 100 *μ*L), nonmarked ligands (nonspecific binding, 100 *μ*L), and 0.2 mL quantitative membrane proteins solution got into the reaction tube. We also added buffer into each tube to make the total reaction volume 0.4 mL. After 15 min incubation in 37°C bath, the reaction stopped in an ice bath. We collected long cell sample on fiberglass filter paper by using a collector and dried it in a drier of 80°C. Then we cut off the sample and measured its radioactivity by using 2000 CA/LL liquid scintillation counter. It was corrected in sample application with blank filter paper. And we used receptor data package (documented by Shanghai Second Medical University) to calculate specific binding count of each point. *B*_max⁡_ (fmol*·*mg) and KD (nmol*·*L) of dopamine receptors were calculated according to Scanchard formulas.

### 2.13. Statistical Analysis

All results are expressed as the means ± SD. The data were analyzed with SPSS 18.0 software.

#### 2.13.1. Statistical Analysis of Neurobehavioral Manifestations

Firstly, Mauchly's test of sphericity was used to judge whether there were relations among the repeated measures data. If any (*P* < 0.05), multivariate ANOVA should be taken next, or Greenhouse-Geisser corrected results should be taken. Treated effect could be evaluated by estimating between-subject variance. Repeated measurement effect or its interactive effect with treated group could be evaluated by estimating within-subject variance. The method of Bonferroni should be used to do pairwise comparisons of the repeated measures data at different measurement time points of each treated group. With multivariate ANOVA, data in different treated group at each measurement time point could be compared pairwise. Differences were considered significant at *P* < 0.05.

#### 2.13.2. Statistical Analysis of Dopamine Receptors Determination

Comparison between two groups was done with independent sample *t*-test and comparison among groups was done with one-way ANOVA analysis. Differences were considered significant at *P* < 0.05. Statistical results were graphed with Excel and SPSS 18.0 software.

### 2.14. Data Mining Based on an Artificial Neural Network BP (BP-ANN)

**Figure 1:**
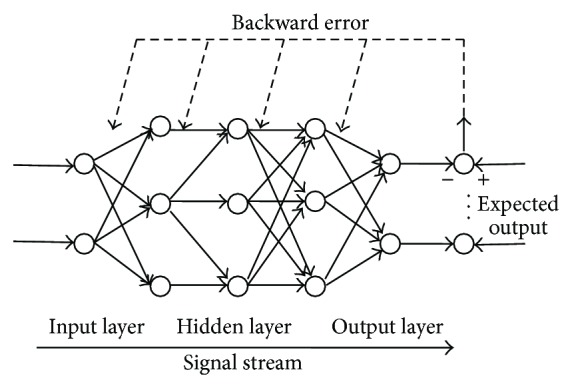
Mathematical description of BP-ANN.

BP-ANN could approximate any nonlinear curve [13]. To identify which factors were associated with the AIM score change and the peak dose rotation change, the network was given a set of inputs and corresponding outputs and determined the parameters of each neuron by analyzing the relationship between the input and output. We selected mRNA of lesion D1 receptor, mRNA of lesion D2 receptor, *B*_max⁡_ of lesion D1 receptor, *B*_max⁡_ of lesion D2 receptor, KD of lesion D1 receptor, and KD of lesion D2 receptor as parameters of the input. Parameters of the output were difference of AIM score and difference of peak dose rotation before and after treatment. After building the model, we could get a stable prediction and judgment model through training and learning of the input samples (as shown in Figure 1).

## 3. Results

### 3.1. AIM Manifestations of LID Rats

See Figure 2.

### 3.2. Comparison of Neurobehavioral Manifestations (AIM Score) before and after Dosing between Different Groups (Shown in Tables 1 and 2 and Figure 3)

ANOVA results of repeated measures data showed that the differences between the groups were statistically significant (*P* < 0.01) (as shown in Table 1), which meant AIM scores were different under different treatment conditions. Time factor was statistically significant (*P* < 0.01), which meant the measurement (AIM score) had a tendency to change over time. The interaction between group and time had obvious statistical significance (*P* < 0.01), which indicated AIM scores at each time point varied with treatment.

**Figure 3:**
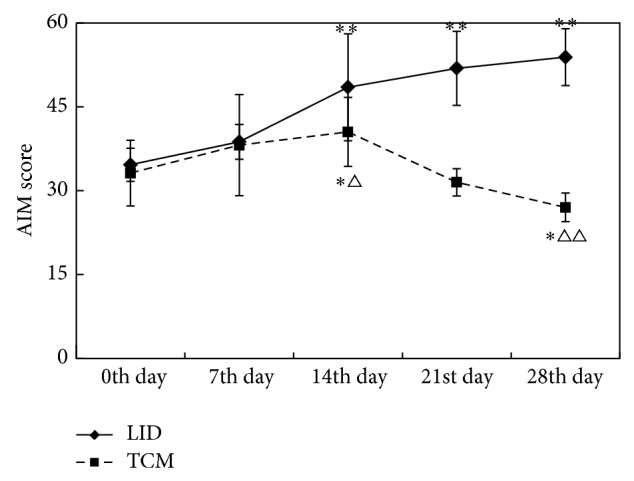
Comparison of AIM score before and after dosing between different groups. (AIM score ^*^ *P* < 0.05, ^**^ *P* < 0.01 compared with 0th day; AIM score ^△^ *P* < 0.05, ^△△^ *P* < 0.01 compared with LID group.)

According to the results of multivariate ANOVA, on the 14th, 21st, and 28th days after treatment, the AIM score in LID group increased progressively (*P* < 0.01) compared with that before treatment (0th day) (as shown in Table 2 and Figure 3). On the 14th day, the AIM score of TCM group increased more than that before treatment (*P* < 0.05). After the 14th day, AIM score decreased progressively and was significantly lower than that before treatment on the 28th day. Since the 14th day, the AIM score of TCM group was lower than that of LID group (*P* < 0.05), but there was no statistical significance between these two groups on the 7th day.

### 3.3. Comparison of Neurobehavioral Manifestations (Peak Dose Rotation) before and after Dosing between Different Groups (Shown in Tables 3 and 4 and Figure 4)

ANOVA results of repeated measures data showed the difference between the groups was statistically significant (*P* < 0.01) (as shown in Table 3), which meant peak dose rotation was different under different treatment conditions. Time factor was statistically significant (*P* < 0.01), which meant the measurement (peak dose rotation) had a tendency to change over time. The interaction between group and time had obvious statistical significance (*P* < 0.01), which indicated peak dose rotation at each time point varied with treatment.

**Figure 4:**
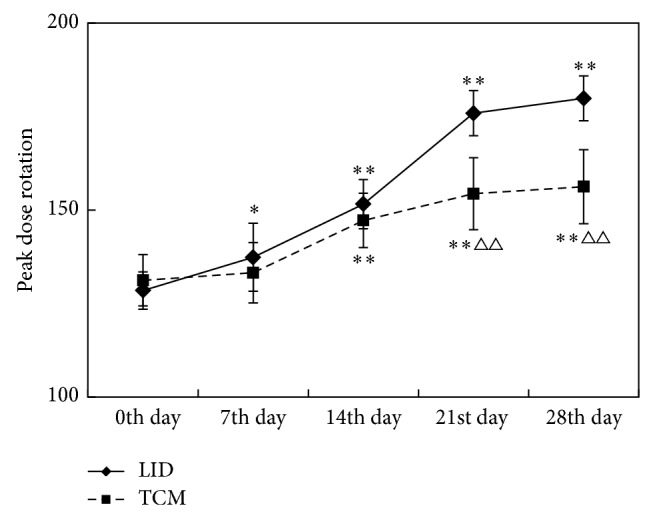
Comparison of peak dose rotation between different groups. (Peak dose rotation ^*^ *P* < 0.05, ^**^ *P* < 0.01 compared with 0th day; peak dose rotation ^△^ *P* < 0.05, ^△△^ *P* < 0.01 compared with LID group.)

According to the results of multivariate ANOVA (as shown in Table 4 and Figure 4), on the 7th, 14th, 21st, and 28th days after treatment, the peak dose rotation in LID group increased progressively (*P* < 0.05) compared with that before treatment (0th day). Since the 14th day, the rotation score of TCM group increased progressively (*P* < 0.01). The rotation score of TCM group was lower than that of LID group since the 14th day (*P* < 0.01).

### 3.4. Comparison of Neurobehavioral Manifestations (LD Efficacy Time) before and after Dosing between Different Groups (Shown in Tables 5 and 6 and Figure 5)

ANOVA results of repeated measures data showed the difference between the groups was statistically significant (*P* < 0.01) (as shown in Table 5), which meant LD efficacy time was different under different treatment conditions. Time factor was statistically significant (*P* < 0.01), which meant the measurement (LD efficacy time) had a tendency to change over time. The interaction between group and time had obvious statistical significance (*P* < 0.01), which indicated LD efficacy time at each time point varied with treatment.

**Figure 5:**
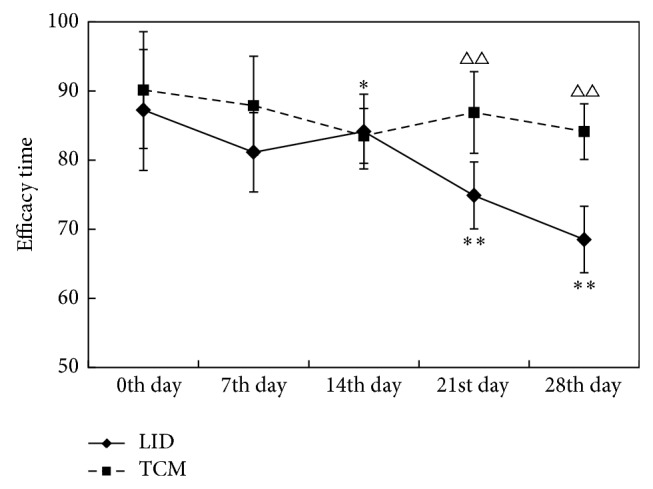
Comparison of LD efficacy time between different groups. (Peak dose rotation ^*^ *P* < 0.05, ^**^ *P* < 0.01 compared with 0th day; peak dose rotation ^△^ *P* < 0.05, ^△△^ *P* < 0.01 compared with LID group.)

According to the results of multivariate ANOVA (as shown in Table 6 and Figure 5), the LD efficacy time in LID group declined (*P* < 0.05) with the treatment time prolonged, compared with that before treatment (0th day). On the 21st day and 28th day, the efficacy time in TCM group was significantly higher than that before treatment (*P* < 0.01). But there was no obvious downward trend in TCM group. The LD efficacy time in TCM group was significantly higher than that in LID model group (*P* < 0.01) on the 21st day and 28th day.

### 3.5. Comparison of Dopamine Receptors Activity in Bilateral Caudate Putamen among Different Groups

#### 3.5.1. Comparison of Dopamine D1 Receptor Activity in Bilateral Caudate Putamen among Different Groups (Shown in Table 7)

The *B*_max⁡_ level of lesion side increased significantly (*P* < 0.01) in the LID group and the TCM group in comparison to that in the control group, and the LID group had the biggest rise. The KD level of lesion side reduced obviously (*P* < 0.05) in the LID group and the TCM group comparing to that in the control group. The *B*_max⁡_ level decreased and the KD level increased (*P* < 0.01) in the TCM group compared to those in the LID group. Comparing to the control group, the *B*_max⁡_ level of normal side increased and KD level decreased in the LID group (*P* < 0.01). The *B*_max⁡_ of normal side in the TCM group increased (*P* < 0.01), and other indexes had no evident differences. The *B*_max⁡_ levels of lesion side were higher and the KD levels were lower than those of normal side in the LID group and the TCM group (*P* < 0.01).

#### 3.5.2. Comparison of Dopamine D2 Receptor Activity in Bilateral Caudate Putamen among Different Groups (Shown in Table 8)

The *B*_max⁡_ level of lesion side decreased significantly (*P* < 0.01) in the LID group and the TCM group in comparison to that in the control group, and the LID group had the biggest falls (*P* < 0.01). The KD level of lesion side significantly elevated (*P* < 0.01) in the LID group comparing to that in the control group. The *B*_max⁡_ level increased and the KD level reduced (*P* < 0.01) in the TCM group compared to those in the LID group (*P* < 0.01). Comparing to the control group, the *B*_max⁡_ level of normal side decreased and KD level increased in the LID group (*P* < 0.05), and other indexes had no evident differences. The *B*_max⁡_ levels of normal side were lower and the KD levels were higher than those of lesion side in the LID group (*P* < 0.01). And the *B*_max⁡_ of lesion side was lower than that of normal side in the TCM group (*P* < 0.01).

### 3.6. Comparison of Gene Expression of Dopamine Receptors in Bilateral Caudate Putamen among Different Groups (Shown in Table 9 and Figures 6 and 7)

#### 3.6.1. Comparison of mRNA Expression of Dopamine D1 Receptor in Bilateral Caudate Putamen among Different Groups

The mRNA expression of dopamine D1 receptor of lesion side increased significantly (*P* < 0.05) in the LID group and the TCM group in comparison to that in the control group, and the LID group had the biggest rise. The mRNA expression reduced (*P* < 0.01) in the TCM group comparing to that in the LID group. Comparing to the control group, the mRNA of normal side showed a significant rise in the LID group and the TCM group (*P* < 0.05), and others had no evident differences. The expression of lesion side was higher than that of normal side in the LID group (*P* < 0.05).

#### 3.6.2. Comparison of mRNA Expression of Dopamine D2 Receptor in Bilateral Caudate Putamen among Different Groups

The mRNA expression of dopamine D2 receptor of lesion side declined obviously (*P* < 0.05) in the LID group and the TCM group in comparison to that in the control group, and the LID group had the biggest drop. The mRNA expression was higher (*P* < 0.01) in the TCM group than that in the LID group. Comparing to the control group, the mRNA expression of normal side showed a significant dip in the LID group (*P* < 0.01), and others had no obvious differences. The expression of lesion side was markedly lower than that of normal side in the LID group and the TCM group (*P* < 0.01).

### 3.7. Main Indicators Affecting AIM Score in the Two Groups Mining by ANN

#### 3.7.1. Relative Importance of Contribution of Input Variables to Output Attribute Values (Difference of AIM Score) in the LID Group

From Table 10, we can see the relative significance of inputs on the output. In the input attribute values, mRNA of D1 lesion side occurred 3 times and the total score was 207.67. And mRNA of D2 lesion side also occurred 3 times and the total score was 171.14. Therefore, in the LID group, the main factors influencing the AIM difference value, in order, were mRNA of D1 lesion side and mRNA of D2 lesion side.

#### 3.7.2. Relative Importance of Contribution of Input Variables to Output Attribute Values (Difference of AIM Score) in the TCM Group

In the input attribute values, mRNA of D2 lesion side occurred 5 times and the total score was 419.56. And mRNA of D1 lesion side also occurred 5 times and the total score was 365.54. Then, the main factors influencing the AIM difference value, in order, were mRNA of D2 lesion side and mRNA of D1 lesion side (as depicted in Table 11).

### 3.8. Main Indicators Affecting Peak Dose Rotation Difference in the Two Groups Mining by ANN

#### 3.8.1. Relative Importance of Contribution of Input Variables to Output Attribute Values (Difference of Peak Dose Rotation) in the LID Group

As shown in Table 12, in the input attribute values, KD of D2 lesion side, mRNA of D1 lesion side, and mRNA of D2 lesion side occurred 5 times, 3 times, and 3 times, respectively. And the total scores of mRNA of D1 lesion side and mRNA of D2 lesion side are 236.65 and 234.7, respectively. Therefore, in the LID group, the main factors influencing the peak dose rotation difference were KD of D2 lesion side, followed by mRNA of D1 lesion side and mRNA of D2 lesion side.

#### 3.8.2. Relative Importance of Contribution of Input Variables to Output Attribute Values (Difference of Peak Dose Rotation) in the TCM Group

As shown in Table 13, in the input attribute values, mRNA of D2 lesion side, mRNA of D1 lesion side, and *B*_max⁡_ of D1 lesion side all occurred 4 times. And the sums of significant scores are 376.54, 234.85, and 180.38, respectively. Therefore, in the TCM group, the main factors influencing the peak dose rotation difference were mRNA of D2 lesion side, followed by mRNA of D1 lesion side and *B*_max⁡_ of D1 lesion side.

## 4. Discussion

At present, the mechanism of LID is not clear yet. Some scholars [14, 15] have proposed that LID is due to the abnormal activity of basal ganglia-thalamus-cortex circuit caused by the imbalance between direct pathway (excitability) mediated by dopamine D1 receptor and indirect pathway (suppressant) mediated by dopamine D2 receptor. Some others [16] believed that activation of direct pathway mediated by dopamine D1 receptor might cause LID.

The results suggested that, with the extension of LD intraperitoneal injection time, the AIM score and peak dose rotation of rats in the LID model group increased progressively, and the symptoms of LID went worse. At the same time, the LD efficacy time shortened gradually with the LD treatment time prolonged. It was because peripheral pharmacokinetics of LD changed, LD elimination increased, its plasma half-life shortened, and bioavailability decreased, which were consistent with “wearing-off” phenomenon happening to PD patients in clinic [17]. It was found that the activity of dopamine D1 receptor degraded significantly, gene expression was upregulated, and the indirect pathway was inhibited. So we thought that LID is due to not only the activation of direct pathway mediated by dopamine D1 receptor, but also the downregulation (reducing to a certain threshold that induces the overinhibition of indirect pathway) in gene expression and activity of the striatal dopamine D2 receptor caused by LD long-term treatment. The inhibition of basal ganglia indirect pathway, which involves projections from the striatum to GPe and from GPe to the STN, leads to the excitation reduction of projections from STN to GPi and SNr. And the inhibition of projections from GPi and SNr to thalamus decreasing finally results in the increase in excitation of thalamus and striatum [18, 19]. When the direct pathway activates, neurotransmitter GABA released by the striatum increased and inhibition to GPi and SNr improves. The decrease in inhibition to thalamus makes a rise in excitatory neurotransmitter Glu [20]. And it finally caused symptoms of LID [21]. The results also indicated that, with the extension of LD treatment time, the normal side striatum also had a rise in activity and affinity of D1 receptor, and the gene expression upregulated. And the activity and affinity of D2 receptor declined, and the gene expression downregulated. This illustrated that there might be a bilateral cross dominant phenomenon in dopaminergic neurons of rats. After damage on one side of dopaminergic pathway by 6-OHDA, the cross dominant fiber of contralateral caudate putamen was also damaged.

The artificial neural network is an application similar to the structure of the brain synaptic connections to the mathematical model of information processing. It focuses on extracting the part of the available features to solve the nonlinear problem that computer or other systems cannot solve. Through the analysis of various influence factors, corresponding mathematical model is established to find the best solution by getting the optimal result [22, 23]. The advantage of ANN is avoiding the model error without hypothesis model of drug behavior [24]. We used BP-ANN to analyze the correlation between neurobehavioral manifestations and activity and gene expression of striatal dopamine D1 and D2 receptors of rats with LID. And we excavated that gene expression of dopamine D1 and D2 receptors was the main factor on AIM score and peak dose rotation of rats with LID and provided a direction for the future research on LID mechanism.

The late Professor Jianhua Hu, Shanghai famous specialist of TCM, extracted Zishenpingchan granule to treat PD on basis of TCM theory, with reference to nonprescription medicine and TCM literature, after decades of exploration and validation. Professor Hu believed that the pathogenesis of PD was deficiency in origin and excess in superficiality. Deficiency refers to the liver and kidney loss and disorder of viscera function. Excess refers to the collection of wind, fire, phlegm, and blood stasis leading to obturation of brain. So nourishing liver and kidney and unblocking collaterals and relieving toxin were the basic treatment. We confirmed in clinical research that TCM had the function of increasing effect and decreasing toxicity [25]. In previous experimental study, we found Zishenpingchan granule could improve the rotation of PD rats by eliminating oxygen free radicals [26], increasing the tyrosine hydroxylase in rats and its mRNA expression [27] and restraining dopamine neurons apoptosis [28]. These mechanisms were closely related to LID.

With the extension of LD treatment time, LD efficacy time shortened gradually in the LID group and the change tendency of efficacy time in the TCM group was not obvious, which meant TCM could prolong LD efficacy time, improved motor fluctuations, and extended the duration of “on-time.” In the TCM group, the activity of dopamine D1 receptor degraded and gene expression was downregulated and the activity of dopamine D2 receptor increased and gene expression was upregulated, comparing to those in the LID group. The results of ANN showed that TCM lowered AIM score and peak dose rotation of rats by improving the gene expression of dopamine D1 and D2 receptors and reducing the imbalance of receptors. This proved that TCM had multitargets effect. But it significantly improved LID neurobehavioral manifestations indicators mainly through upregulating dopamine D2 receptor gene expression. TCM inhibited the overactivation in direct pathway by decreasing the activity of dopamine D1 receptor and downregulating the gene overexpression. We could find that TCM had a similar effect as D1 receptor blockers. After treatment of TCM, the activity of dopamine D2 receptor rose, gene expression was upregulated, and the inhibition of indirect pathway was reduced. TCM also had a similar effect as D2 receptor agonist. So we thought that TCM prolonged LD efficacy time and effectively relieved LID symptoms through regulating the gene expression and activity of dopamine D1 and D2 receptors, improving the imbalance of direct pathway and indirect pathway and reducing the volatility stimulation of LD on postsynaptic membrane.

## Figures and Tables

**Figure 2:**
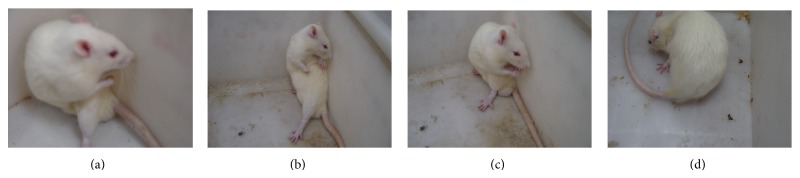
AIM manifestations of LID rats. (a) Limb movement disorder. (b) Axiality movement. (c) Chewing. (d) Contralateral rotation.

**Figure 6:**
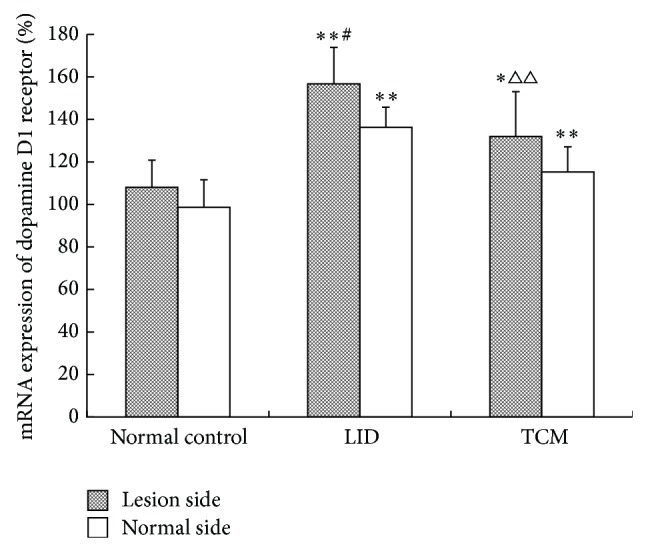
Comparison of the relative quantitative mRNA expression of dopamine D1 receptor in bilateral caudate putamen among different groups.

**Figure 7:**
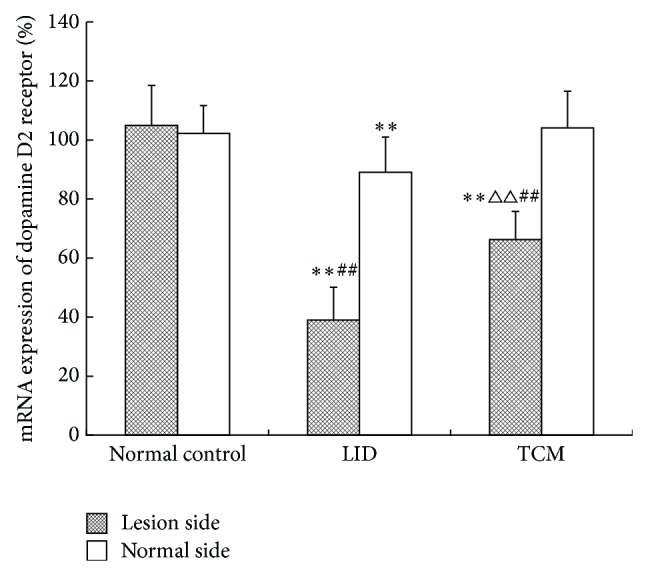
Comparison of the relative quantitative mRNA expression of dopamine D2 receptor in bilateral caudate putamen among different groups. (^*^ *P* < 0.05, ^**^ *P* < 0.01 compared with normal control group; ^△^ *P* < 0.05, ^△△^ *P* < 0.01 compared with LID group; ^#^ *P* < 0.05, ^##^ *P* < 0.01 versus normal side.)

**Table 1:** ANOVA results of repeated measures data of AIM score.

Source of variation	SS	DF	MS	*F*	*P*
Variation within group					
Time	1008.33	2.64	382.04	13.93	<0.01
Time*·*group	2182.68	2.64	826.98	30.16	<0.01
Error	1013.40	36.95	27.43	—	—
Variation between groups					
Group	2633.51	1	2633.51	26.20	<0.01
Error	1407.48	14	100.53	—	—

**Table 2:** Comparison of AIM score before and after dosing between different groups.

Group	*n*	Time point				
		0th day	7th day	14th day	21st day	28th day
LID	8	34.63 ± 2.97	38.75 ± 3.11	48.50 ± 9.55^**^	51.88 ± 6.60^**^	53.88 ± 5.06^**^
TCM	8	33.13 ± 5.87	38.13 ± 9.05	40.50 ± 6.19^△^	31.50 ± 2.45^△△^	27.00 ± 2.56^△△^

AIM score ^*^ *P* < 0.05, ^**^ *P* < 0.01 compared with 0 Day; AIM score ^△^ *P* < 0.05, ^△△^ *P* < 0.01 compared with LID group.

**Table 3:** ANOVA results of repeated measures data of peak dose rotation.

Source of variation	SS	DF	MS	*F*	*P*
Variation within group					
Time	18801.88	1.74	10814.96	132.02	<0.01
Time*·*group	2185.83	1.74	1257.30	15.35	<0.01
Error	1933.90	24.34	81.92	—	—
Variation between groups					
Group	2070.61	1	2070.61	14.16	<0.01
Error	2047.48	14	146.25	—	—

**Table 4:** Comparison of peak dose rotation between different groups.

Group	*n*	Time point				
		0th day	7th day	14th day	21st day	28th day
LID	8	128.50 ± 4.96	137.38 ± 9.09^*^	151.63 ± 6.57^**^	175.88 ± 6.03^**^	179.88 ± 5.99^**^
TCM	8	131.25 ± 6.90	133.25 ± 8.05	147.25 ± 7.27^**^	154.38 ± 9.61^∗∗△△^	156.25 ± 9.87^∗∗△△^

Peak dose rotation ^*^ *P* < 0.05, ^**^ *P* < 0.01 compared with 0 Day; peak dose rotation ^△^ *P* < 0.05, ^△△^ *P* < 0.01 compared with LID group.

**Table 5:** ANOVA results of repeated measures data of LD efficacy time.

Source of variation	SS	DF	MS	*F*	*P*
Variation within group					
Time	1349.83	4	337.46	8.04	<0.01
Time*·*group	696.33	4	174.08	4.15	<0.01
Error	2349.85	56	41.96	—	—
Variation among groups					
Group	1073.11	1	1073.11	55.28	<0.01
Error	271.78	14	19.41	—	—

**Table 6:** Comparison of LD efficacy time between different groups.

Group	*n*	Time point				
		0th day	7th day	14th day	21st day	28th day
LID	8	82.75 ± 8.75	81.13 ± 5.74	84.13 ± 5.41	74.88 ± 4.85^**^	68.50 ± 4.81^**^
TCM	8	90.13 ± 8.44	87.88 ± 7.16	83.50 ± 3.96^*^	86.38 ± 5.89^△△^	84.13 ± 4.02^△△^

Peak dose rotation ^*^ *P* < 0.05, ^**^ *P* < 0.01 compared with 0 Day; peak dose rotation ^△^ *P* < 0.05, ^△△^ *P* < 0.01 compared with LID group.

**Table 7:** Comparison of dopamine D1 receptor activity in bilateral caudate putamen among different groups ($\overset{-}{x}\pm s$).

Group	*n*	*B*_max⁡_ (fmol/mg)		KD (nmol/L)	
		Lesion side	Normal side	Lesion side	Normal side
Normal control	8	1118.99 ± 86.84	1075.22 ± 90.92	2.499 ± 0.289	2.554 ± 0.216
LID	8	1653.63 ± 59.88^∗∗##^	1440.80 ± 55.37^**^	0.730 ± 0.035^∗∗##^	1.858 ± 0.126^**^
TCM	8	1390.97 ± 52.40^∗∗△△##^	1306.00 ± 51.64^**^	1.342 ± 0.063^∗∗△△##^	2.485 ± 0.197

^*^ *P* < 0.05, ^**^ *P* < 0.01 compared with normal control group; ^△^ *P* < 0.05, ^△△^ *P* < 0.01 compared with LID group; ^#^ *P* < 0.05, ^##^ *P* < 0.01 compared with normal side.

**Table 8:** Comparison of effect of TCM on dopamine D2 receptor activity in bilateral caudate putamen among different groups ($\overset{-}{x}\pm s$).

Group	*n*	*B*_max⁡_ (fmol/mg)		KD (nmol/L)	
		Lesion side	Normal side	Lesion side	Normal side
Normal control	8	1284.87 ± 155.54	1246.71 ± 124.45	1.021 ± 0.107	0.996 ± 0.120
LID	8	533.16 ± 44.14^∗∗##^	1075.54 ± 77.00^*^	1.587 ± 0.050^∗∗##^	1.204 ± 0.075^**^
TCM	8	771.85 ± 103.00^∗∗△△##^	1194.03 ± 141.67	1.067 ± 0.070^△△^	1.097 ± 0.124

^*^ *P* < 0.05, ^**^ *P* < 0.01 compared with normal control group; ^△^ *P* < 0.05, ^△△^ *P* < 0.01 compared with LID group; ^#^ *P* < 0.05, ^##^ *P* < 0.01 compared with normal side.

**Table 9:** Comparison of the relative quantitative mRNA expression of dopamine D1 and D2 receptors in bilateral caudate putamen among different groups.

Group	*n*	D1 receptor		D2 receptor	
		Lesion side	Normal side	Lesion side	Normal side
Normal control	8	1.081 ± 0.128	0.987 ± 0.129	1.049 ± 0.136	1.022 ± 0.095
LID	8	1.567 ± 0.172^∗∗#^	1.363 ± 0.095^**^	0.390 ± 0.111^∗∗##^	0.891 ± 0.119^**^
TCM	8	1.320 ± 0.211^∗△△^	1.153 ± 0.118^**^	0.663 ± 0.095^∗∗△△##^	1.041 ± 0.124

^*^ *P* < 0.05, ^**^ *P* < 0.01 compared with normal control group; ^△^ *P* < 0.05, ^△△^ *P* < 0.01 compared with LID group; ^#^ *P* < 0.05, ^##^ *P* < 0.01 versus normal side.

**Table 10:** Relative importance of contribution of input variables to output attribute values in LID group (difference of AIM score) in the LID group.

Score	The significance of output attribute values (difference of AIM score)		
Input attribute values	<15	15–17.67	17.67–22.47
mRNA of D1 lesion side	53.94	100	53.73
mRNA of D2 lesion side	38.68	66.23	66.23
*B*_max⁡_ of D1 lesion side	—	—	100
*B*_max⁡_ of D2 lesion side	—	—	—
KD of D1 lesion side	58.41	—	—
KD of D2 lesion side	—	60.13	—

**Table 11:** Relative importance of contribution of input variables to output attribute values in LID group (difference of AIM score) in the TCM group.

Score	The significance of output attribute values (difference of AIM score)							
Input attribute values	−19	−11	−10	−6	−5	−2	0	4
mRNA of D1 lesion side	79.90	94.99	82.58	35.28	—	72.79	—	—
mRNA of D2 lesion side	99.82	—	72.45	—	—	73.70	100	73.59
*B*_max⁡_ of D1 lesion side	—	48.83	—	31.13	86.70	—	—	74.38
*B*_max⁡_ of D2 lesion side	100	—	—	—	84.83	—	—	—
KD of D1 lesion side	—	33.38	—	30.98	—	—	87.09	—
KD of D2 lesion side	—	—	100	—	—	100	93.29	73.19

**Table 12:** Relative importance of contribution of input variables to output attribute values (difference of peak dose rotation) in the LID group.

Score	The significance of output attribute values (peak dose rotation difference)				
Input attribute values	11	17	19	21	23
mRNA of D1 lesion side	100	89.80	—	46.85	—
mRNA of D2 lesion side	—	100	54.57	—	80.13
*B*_max⁡_ of D1 lesion side	—	—	70.56	—	86.83
*B*_max⁡_ of D2 lesion side	77.77	—	—	74.22	—
KD of D1 lesion side	—	—	—	—	—
KD of D2 lesion side	88.77	96.86	50	100	84.66

**Table 13:** Relative importance of contribution of input variables to output attribute values (peak dose rotation difference) in the TCM group.

Score	The significance of output attribute values (peak dose rotation difference)			
Input attribute values	−19~−10.67	−10.67~−6.13	−6.13~−1.58	−1.58~4.00
mRNA of D1 lesion side	36.07	48.12	74.55	76.11
mRNA of D2 lesion side	100	76.54	100	100
*B*_max⁡_ of D1 lesion side	24.91	32.01	59.65	63.81
*B*_max⁡_ of D2 lesion side	—	—	—	—
KD of D1 lesion side	—	—	—	—
KD of D2 lesion side	—	—	—	—
